# Unravelling the nonlinear generation of designer vortices with dielectric metasurfaces

**DOI:** 10.1038/s41377-025-01741-0

**Published:** 2025-01-16

**Authors:** Laure Coudrat, Guillaume Boulliard, Jean-Michel Gérard, Aristide Lemaître, Aloyse Degiron, Giuseppe Leo

**Affiliations:** 1 Laboratoire Matériaux et Phénomènes Quantiques, Université Paris Cité and CNRS, Paris, 75013 France; 2 Université Grenoble Alpes, CEA, INP, IRIG, PHELIQS, Grenoble, 38000 France; 3 Centre de Nanosciences et de Nanotechnologies, CNRS - Université Paris-Saclay, Palaiseau, 91120 France; 4 Institut universitaire de France (IUF), Paris, France

**Keywords:** Metamaterials, Nanophotonics and plasmonics

## Abstract

Vortex beams are currently drawing a great deal of interest, from fundamental research to several promising applications. While their generation in bulky optical devices limits their use in integrated complex systems, metasurfaces have recently proven successful in creating optical vortices, especially in the linear regime. In the nonlinear domain, of strategic importance for the future of classical and quantum information, to date orbital angular momentum has only been created in qualitative ways, without discussing discrepancies between design and experimental results. Here, we demonstrate and analyze the generation of high-purity second harmonic (SH) optical vortices via dielectric meta-holograms. Through full-wave simulations and a proper fabrication protocol, we achieve efficient frequency doubling of an unstructured pump beam into SH vortices with topological charges from 1 to 10. Interferometric and modal-purity measurements confirm the generation of high-quality SH vortices with minimal deviations from the intended design thanks to a quasi-local control over the SH phase. Through systematic comparisons between experimental data and semi-analytical calculations, we also provide a clear insight into the occurrence of ghost vortices in the metasurface-generated harmonic beams, highlighting the importance of simple designs that can be readily transposed into fabricated devices with high fidelity. Our findings underscore the potential of nonlinear dielectric metasurfaces for versatile structured-light generation and manipulation, paving the way for future developments in integrated photonic systems.

## Graphical abstract

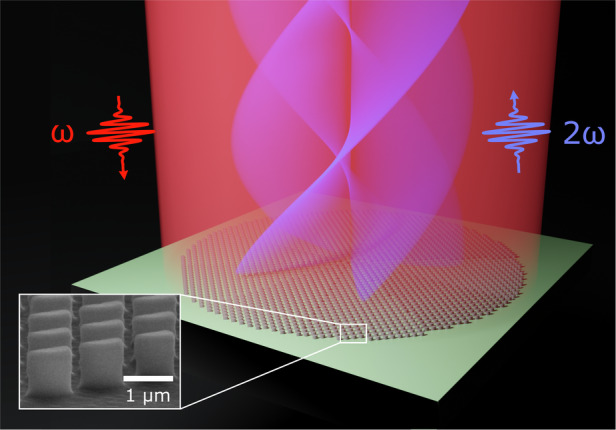

## Introduction

Structured light^1^, which denotes optical fields with non-trivial phase and/or polarization textures, plays an ever-increasing role in physical and life sciences^2^. One of the most emblematic classes of structured light is formed by optical vortices, that is, beams with helical wavefronts. The phase of a vortex, when plotted in a plane perpendicular to the propagation direction, varies $m$ times between 0 and 2π around a central singularity, with $m$ a non-zero integer or fractional value called the topological charge that quantifies the number and winding direction of the helical wavefronts. This peculiar phase texture is associated with an orbital angular momentum (OAM), equal to $m\hbar$^3,4^, which offers extra degrees of freedom for optical tweezers, metrology^5^, optical communications^6,7^, and quantum optics^8^. Moreover, the phase singularity along the propagation direction implies a null in intensity at the center of the beam. This typical annular shape is the key ingredient of stimulated-emission-depletion microscopy^9,10^, a celebrated fluorescent imaging technique that provides spatial resolution well below the diffraction limit.

There exists a variety of techniques to structure light into optical vortices. Most of them rely on the interaction of a spatially coherent beam with macroscopic optical elements such as cylindrical lenses^11^, spatial light modulators^12^ and phase plates^13^, or on spin-orbit coupling effects that arise in focused laser beams^14^. These developments laid the groundwork for more advanced manipulations of light fields. Further progress concerned the nonlinear regime^15^, with notable demonstrations of OAM algebra in SH^16^ and sum frequency generation^17^, and nonlinear spin-orbit coupling in both bulk crystals^18,19^ and thin films^20^.

Optical vortices have also been successfully generated with metasurfaces^21–26^, i.e. optically thin planar patterns made of subwavelength inclusions and/or diffractive elements. Metasurfaces are advantageous for their compact size, lightweight, and compatibility with semiconductor fabrication techniques, which makes them ideal candidates for integrating structured light functionalities into advanced photonic systems. They can tailor the wavefront of incident light using geometric (also known as Pancharatnam-Berry, PB) phase, guidance, or resonant-phase (Huygens) approaches. With the PB method, the phase front is modulated by rotating each nanostructure, whereas the guidance and resonant phase methods involve variations in the shape or size of each nanostructure, to modify the effective index of vertically guided modes and excite electric and magnetic dipoles, respectively. Importantly, in the guidance approach, which typically yields high diffraction efficiencies^27^, the 0-2π phase excursion requires structures with higher aspect ratios than their PB and Huygens counterparts, thus imposing more stringent technological challenges for low-cost and large-scale manufacturing.

Both Huygens and PB principles have been extended to the nonlinear regime, leading to the demonstration of high harmonic generation^28–30^, harmonic beam shaping^31–36^ and nonlinear vortex beam generation at the second^37–39^ and third^31,40^ harmonics. To date, most vortices observed at the SH have been generated with plasmonic metasurfaces, with a focus on small topological charges and typically neither an analysis of their purity nor a quantitative comparison with the intended design. Vortices generated at the third harmonic were instead obtained with high-contrast dielectric metasurfaces made of technologically mature semiconductor materials with large energy gaps (e.g. silicon in refs. ^31,40^). Despite a weaker field confinement, this material choice proved advantageous with respect to plasmonic or hybrid plasmonic-dielectric platforms due to higher nonlinearities, lower losses and high damage thresholds, especially in the visible and near-infrared range. While topological charges as high as 16 were observed^40^, the purity of third-harmonic vortices and their experimental deviations with respect to the design do not seem to be more discussed in the literature than for SH vortices.

In this work, based on a simple fabrication protocol, we propose a nonlinear metasurface platform capable of generating high-quality SH vortices with a large span of topological charges. Experimental features, down to the smallest details of their phase and intensity profiles, are in remarkable quantitative agreement with the design. The metasurfaces, consisting of Aluminum Gallium Arsenide (AlGaAs) asymmetric pillars placed on an aluminum oxide (AlOx) substrate, give rise to highly directional on-axis SH emission whose local phase is precisely encoded through the sound control on the transverse dimensions of each pillar. In this framework, we generate SH vortices with topological charges from 1 to 10. We perform an extensive experimental characterization of their experimental phase profiles and modal purities and explain our results with a semi-analytical model that captures all the salient characteristics of the generated beams. Such nonlinear dielectric metasurfaces constitute an integrated platform for versatile nonlinear structured light generation and manipulation, with potential impact in optical communications and holographic imaging.

## Results

### Numerical design of nanoresonators and metasurfaces

Our nonlinear metasurfaces consist of periodic arrangements of [100] Al_0.18_Ga_0.82_As resonators on an AlOx substrate. These dielectric resonators have been designed with full-wave simulations in COMSOL Multiphysics (see Materials and methods) to satisfy multiple demanding conditions simultaneously. First, the choice of materials was made to enable optical resonances (Fig. S4) with tight electromagnetic field confinement at both the fundamental frequency (FF) and SH wavelengths (λ_FF_ = 1550 nm, λ_SH_ = 775 nm) by taking advantage of the high contrast between the refractive indices of AlGaAs alloys (*n* > 3)^41^ and aluminum oxide (*n* = 1.6)^42^. This confinement, combined with the strong second-order susceptibility tensor of AlGaAs alloys^43^, leads to efficient frequency-doubled signals and minimizes crosstalk between neighboring elements. Second, the geometry of the resonators has been chosen to ensure directional SH emission normal to the surface. Cylindrical [100] AlGaAs resonators are characterized by radiation diagrams with lobes at large angles and typically zero on-axis emission (see Fig. S1). In the literature, redirection of the SH emission towards the normal direction was successfully accomplished through several approaches including tilted excitation relative to the metasurface^44^, alternative crystalline orientations^45^, engineered array factors^46^ and symmetry breaking of the resonator shape^34,47^. In refs. ^34,47^, the AlGaAs resonators with broken symmetry consisted of half-elliptic cylinders on top of elliptical pillars, and the fabrication of such nanochairs required two steps of electron beam lithography (EBL).

**Fig. 1:**
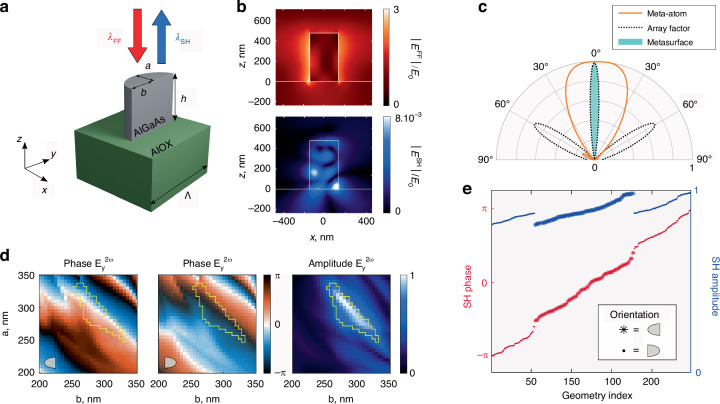
Nonlinear metasurface modeling. **a** Sketch of the nanoresonator. **b** Electric near-field distribution at ${\lambda }_{{FF}}=1550$ nm (top) and ${\lambda }_{{SH}}=775$ nm (bottom). The amplitude has been normalized to the pump amplitude ${E}_{0}$. **c** SH radiation diagram of a half-elliptical resonator with $a=b=280$ nm. **d** Look-up tables of the SH phase (left and middle) and normalized SH amplitude (right) versus $a$ and $b$. Each calculation has been made with a single unit-cell flanked by periodic boundary conditions, to take into account the small crosstalk between neighboring elements. The left and middle panels correspond to two π -rotated orientations of the half-elliptical pillars, as sketched in the bottom left corner. **e** SH phase (red markers) and normalized amplitude (blue markers) as a function of a meta-atom geometry index corresponding to ordered ($a,b$) pairs with full 0–2 π range and flat amplitude, highlighted with a yellow perimeter in (**c**)

In this work, we employ a similar design strategy, with a crucial modification. We simply consider half-elliptic cylinders (Fig. 1a), for which our simulations still predict a pronounced strong confinement of the fields in the volume of the resonator (Fig. 1b) as well as on-axis SH emission lobe under normal pump incidence (Fig. 1c), with the additional fabrication simplification of requiring a single EBL step (see below as well as Materials and Methods). Moreover, the maps of Fig. 1d show that the phase and amplitude of the on-axis SH far-field can be tuned with considerable breadth by varying the major and minor semiaxes $a$ and $b$ of the resonators. Each point of these maps represents the result of far-field calculations versus $a$ and $b$, for a height *h* = 476 nm, and two π-rotated orientations of the pillars (see Fig. S3). The large and complex variations from one pixel to another reflect the resonant nature of our meta-atoms, where the variation of the above parameters impacts on nonlinear interactions between FF and SH modes. In particular, the phase can be varied from 0 to 2π, making it possible to construct metasurfaces of half-elliptic AlGaAs resonators emitting SH light with arbitrary phase profiles.

Let us now use this library of resonators to design a nonlinear metasurface that generates SH vortex beams of topological charge $m$. In principle, such beams can be obtained with a metasurface that emits SH light with the same amplitude at every point and with a phase that varies radially $m$-times between 0 and 2π (see Materials and methods and Supplementary, Fig. S11). We discretize these ideal amplitude and phase profiles into pixels having the same size as the period $\varLambda$ of the targeted metasurface ($\varLambda$ = 900 nm, chosen to minimize the crosstalk between the resonators while being small enough to sample the intended phase profile correctly). Finally, we select among the library of meta-atoms of Fig. 1d a subset of geometries whose predicted SH far-field properties are as close as possible to those of the discretized profile and replace each pixel by the selected resonator. We select 247 resonators with a normalized amplitude close to a targeted optimal value and with the best on-axis directionality, so as to maximize SH generation and collection. This subset of resonators is represented in Fig. 1e.

### Experimental results and comparison with semi-analytical modeling

**Fig. 2:**
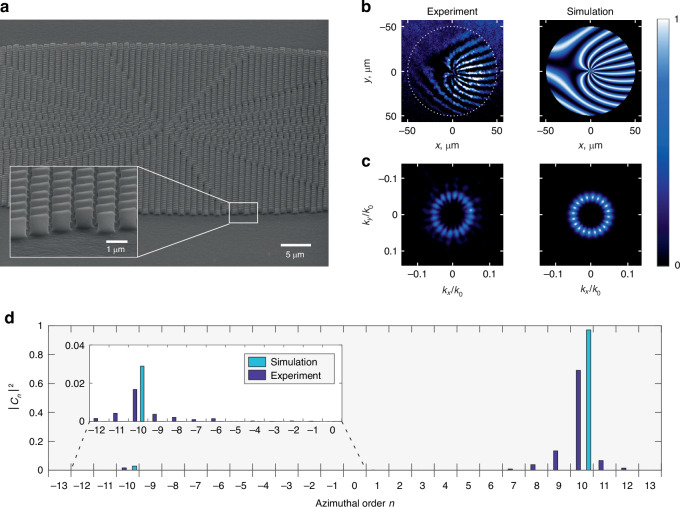
Characterization of the SH vortex beam with $$m$$$$=$$$$10$$. **a** SEM image of the metasurface. **b** Experimental (left) and simulated (right) real-space SH interference pattern. **c** Experimental (left) and simulated (right) SH intensities in the Fourier plane. **d** Modal analysis of the vortex beam emitted by the metasurface. Modal weights $${\left|{c}_{n}\right|}^{2}$$ are associated to the $$L{G}_{0,n}$$ modes. As discussed in the text and the Supplementary Section 3.3, the contribution of the $$L{G}_{0,m\pm \mathrm{1,2}}$$ modes is overestimated in the experiments

To assess the potential of our platform to tailor the SH wavefront, we designed, fabricated and characterized a device that generates a vortex beam with topological charge $m=10$ at the SH. In this case, 172 different types of resonators of Fig. 1e were used to implement the desired phase profile. The fabrication, detailed in Materials and methods, involved the transfer of the half-elliptical shapes of the resonators in a layer of HSQ resist spun onto a [100] Al_0.18_Ga_0.82_As-on-Al_0.98_Ga_0.02_As wafer by EBL, followed by inductively coupled-plasma reactive-ion etching and oxidation of the substrate. A scanning electron micrograph of the fabricated metasurface is shown in Fig. 2a. To characterize the sample, we built a versatile optical setup described in Fig. S6. The metasurface is pumped by linearly polarized pulses at λ_FF_ = 1550 nm through a 10X microscope objective that is also used to collect and analyze the SH signal at λ_SH_ = 775 nm. Successful encoding of the SH wavefront is first analyzed with a characterization of the phase texture of the SH beam. Experimentally, we let the SH beam from the device interfere with a reference beam in a generalized Mach-Zehnder configuration. The resulting fork-like interference pattern shown in Fig. 2b possesses a dislocation in its center, revealing the phase singularity associated with the encoded helical phase profile. This measurement is in excellent agreement with a simple model in which each resonator of the metasurface is replaced by a source whose amplitude and phase are those of the numerical design (Fig. 1e). As shown on the right panel of Fig. 2b, the experimental fork-like pattern is well reproduced by the calculations when one makes this composite array of sources interfere with a slightly tilted Gaussian beam (this tilt simply takes into account that the recombined beams of our experimental interferometer are not strictly collinear). Furthermore, not only does the interferogram demonstrate that the metasurface produces a vortex, but it also provides the value of the topological charge $m$ of the OAM carried by the SH beam. This value, $m=10$, can be read directly from the experimental or calculated interference pattern by counting the number of bright fringes around the dislocation. Alternatively, a Fourier transform of the interference pattern provides the added phases of the two beams that interfered. The result of this method is given in Fig. S7, where a helical wave front with $m$ branches is clearly evidenced.

We next analyze the intensity profile of the vortex in the Fourier plane, by imaging the back focal plane of the microscope objective. Figure 2c shows that the measured SH beam displays a necklace pattern, which is well reproduced by our simple model that treats the metasurface as a composite source array emitting light at the SH wavelength. While such bead-like pattern is typical of vortex beams generated by dielectric metasurfaces^26,31,40^, it does not seem to have been commented until very recently, and only in the linear regime, by De Oliveira et al.^26^. These authors showed that imperfections in the fabricated metasurfaces lead to the generation of a ghost vortex with a topological charge opposite to that of the intended OAM. Despite the fact that this ghost vortex has a much weaker intensity than the main one, the two beams interfere together. The necklace pattern is the result of this interference.

The same reasoning can be applied for our nonlinear beams. In general, the interference of two vortices with topological charges $m$ and $m\mbox{'}$ produces a pattern with $\left|m-m\mbox{'}\right|$ maxima^48,49^. We have already demonstrated that the main nonlinear vortex produced by our metasurface has a topological charge *m* = 10. Given that we count 20 maxima in the necklace pattern of Fig. 2c, we infer the existence of a *ghost* vortex with topological charge $m^{\prime}=-10$.

This result can be confirmed with purity measurements in which the SH beam is projected on the basis of the zeroth radial order, *n*th azimuthal order Laguerre-Gauss modes ${{\rm{LG}}}_{0,n}$. The contribution of each ${{\rm{LG}}}_{0,n}$ mode is given by a modal weight coefficient ${\left|{c}_{n}\right|}^{2}$ that can be experimentally measured (see Methods). In the purity spectrum, shown in Fig. 2d, the existence of a *ghost* vortex with charge $m\mbox{'}=-10$ is confirmed, with a contribution close to 2%. In comparison, the main peak observed for $n=m=10$ reaches 69%. The experimental purity is lower than that predicted by our simple composite source array model, also shown in Fig. 2d. This discrepancy can be ascribed to the difficulty of accurately measuring the contribution of the $L{G}_{\mathrm{0,10}\pm \mathrm{1,2}}$ modes due to a limited resolution of the detector (see details in Supplementary Section 3.3), which leads to a gross overestimation of their weight in the measurements. To support this claim, we have verified that the necklace intensity pattern with 20 beads of Fig. 2c cannot be reconstructed by taking into account the measured contribution of the $L{G}_{\mathrm{0,10}\pm \mathrm{1,2}}$ modes (Fig. S9). In other words, the only non-negligible mode beside the $m=10$ vortex is the ghost vortex with $m^{\prime} =-10$, which contributes to ~2% to the far-field SH signal.

## Discussion

**Fig. 3:**
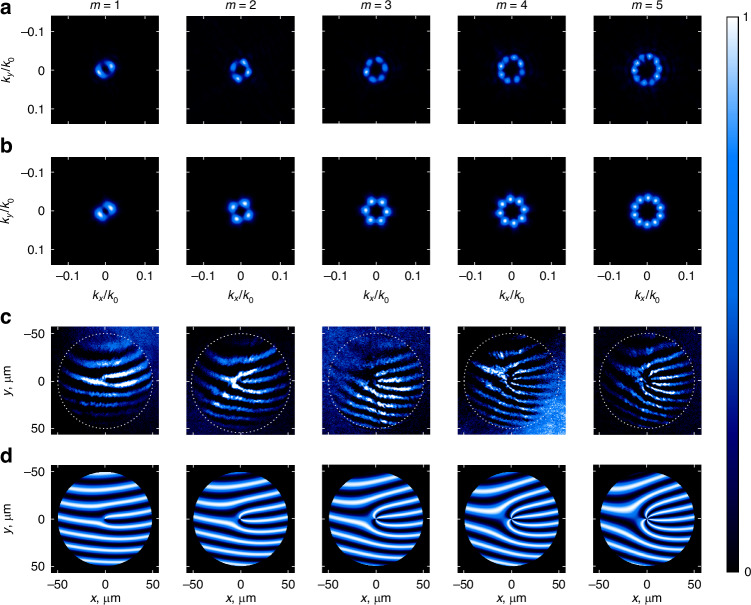
On-demand SH vortex beam generation. **a** Experimental and **b** simulated SH intensities in the Fourier plane. **c** Experimental and **d** simulated real-space SH interference patterns

**Fig. 4:**
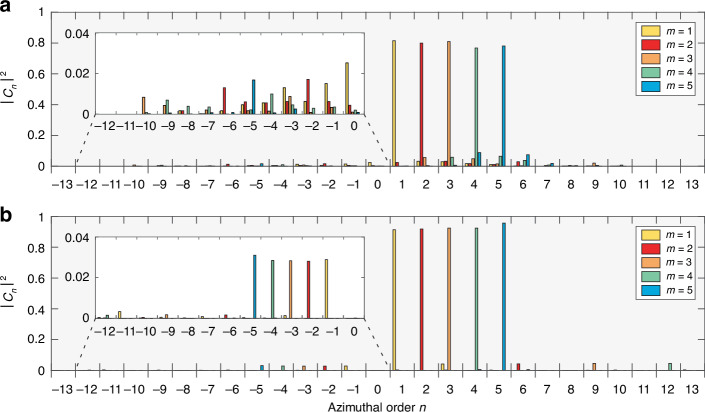
Mode purity analysis. **a** Experimental and **b** simulated modal decomposition of the SH vortices with topological charges *m* from 1 to 5. As before, the contribution of the $L{G}_{0,m\pm \mathrm{1,2}}$ modes is overestimated in the experiments

All the results described above for $m=10$ can be generalized to nonlinear metasurfaces generating SH vortex beams with other topological charges. As an illustration, we present the experimental (Fig. 3a, c) and simulated (Fig. 3b, d) results for metasurfaces generating vortex beams with topological charges from $m=1$ to $m=5$. For all values of $m$, Fourier plane intensities exhibit $2m$ azimuthal undulations (Fig. 3a), indicating the presence of a ghost vortex with charge $m\mbox{'}=-m$. Once again, the topological charge $m$ can also be evaluated through interferometric measurements with the presence of $m$ maxima around a fork dislocation (Fig. 3c). These results were confirmed in all cases by a modal decomposition analysis which revealed a main contribution for $n=m$ and a minor contribution for $n=-m$ (Fig. 4), with an experimental mode purity reaching 80% (as for the $m=10$ case, the actual experimental purity is higher due to the difficulty of accurately measuring the weight of the $L{G}_{0,m\pm \mathrm{1,2}}$ modes).

As discussed by De Oliveira et al. in the linear regime^26^, the presence of a ghost vortex with a topological charge opposed to the main one implies that the metasurface is sensitive to deviations of the ideal phase and amplitude profiles. In our case, the ghost vortex cannot be mainly attributed to fabrication defects since it is also well captured by our semi-analytical model for all the structures presented in this study (we note in passing that this excellent agreement between experiments and calculations demonstrates that the designed structures can be fabricated with an extremely high fidelity). Instead, we ascribe the origin of the ghost vortex to the small variations of the SH generation efficiency from one resonator geometry to another. If, in our semi-analytical model, we impose an equal amplitude to all the sources, the bead pattern in the intensity profile disappears and a single ring characteristic of a vortex with 100% purity is observed (Fig. S11).

Let us mention in passing that all the SH vortex beams generated in this work are experimentally verified to be scalar, their uniform linear polarization state along *y* being dictated by the polarization selection rule of the χ^(2)^ interaction within each meta-atom (Fig. S3). Interestingly, this is at variance with the recent demonstration of vectorial vortex SH generation in an unstructured AlGaAs membrane^19^.

Finally, although the SH generation efficiency is not the main focus of this work, it was precisely measured (see Supplementary Section 3.4) and found to be 50 times larger than in an unstructured AlGaAs membrane^19^and 4 times larger than in previous works on SH wavefront shaping with the same platform^34^. In this respect, let us mention that nonlinear meta-optics has progressively branched into two distinct research focuses: one on the enhancement of the conversion efficiency, and another one on the efficient structuration of harmonic light fields at subwavelength scale. Here we provided a simple design, backed by robust electromagnetic simulations and modeling, to generate non-trivial modes of nonlinear light.

Beyond the scope of this work, further optimization of the nanofabrication protocol and of the resonance quality factors may pave the way towards more efficient generation of highly structured harmonic fields.

## Materials and methods

### Full-wave simulations

SH near fields are simulated in COMSOL Multiphysics with a two-step calculation process. First, a linear study at the pump frequency $\omega$ using the scattered field formulation allows one to extract the total near-field distribution in the resonator volume, for a given analytical background field. Then, the associated nonlinear current distribution is calculated according to ${P}_{i}^{(2\omega )}={{\rm{\varepsilon }}}_{0}\sum _{{jk}}{{\rm{\chi }}}_{{ijk}}^{\left(2\right)}{E}_{j}^{\omega }{E}_{k}^{\omega }$. The latter constitutes a source term for a second study at $2\omega$ that provides the SH near-field distribution. Finally, we use the generalized formulation of Stratton-Chu formula embedded in the RETOP^50^ package to determine the SH far-field properties. In this framework, the nanostructure semi-axes are swept to obtain the look-up table of Fig. 1d, relating the SH phase and amplitude to the resonator geometry.

### Semi-analytical model

To design the metasurface, we calculate the finite-sized transverse phase distribution $\exp \left({im}\theta \right)$ of a vortex beam with topological charge $m$. Such continuous phase profile is discretized with respect to the metasurface period before being mapped to a geometry profile according to our look-up table. Then, we predict the SH beam profile by calculating the far-field pattern of the complex hologram $H\left(x,y\right)=A(x,y)\exp (i\varphi (x,y))$, where $A$ and $\varphi$ are respectively the SH amplitude and phase of the resonator at position $(x,y)$. The simulated interference patterns are calculated according to ${\left|H\left(x,y\right)+E(x,y)\right|}^{2}$, $E$ being a tilted gaussian beam with a finite radius of curvature. The tilt angle is ~3° and reflects the fact that the SH vortex beam and reference Gaussian beams are not strictly collinear upon recombination on the second beamsplitter of our generalized Mach-Zehnder interferometer.

### Metasurface fabrication

The fabrication follows the procedure detailed in refs. ^46,51,52^. It starts with the molecular beam epitaxy of a vertical structure: (100) GaAs / GaAs-to-Al_0.98_Ga_0.02_As buffer (90 nm) / Al_0.98_Ga_0.02_As (2 µm) / Al_0.98_Ga_0.02_As-to-Al_0.18_Ga_0.82_As buffer (90 nm) / Al_0.18_Ga_0.82_As (476 nm). The 100 µm large metasurface is patterned on the Al_0.18_Ga_0.82_As surface by EBL on a 100 nm thick HSQ layer. The adhesion of HSQ to the substrate is promoted by adding a thin layer of Surpass 3000 before spinning the e-beam resist. EBL is performed in a Raith Pioneer II system with 20 kV acceleration voltage and 1 mC/cm² exposure dose. The resist is developed in 1:3 MIBK/H_2_O solution. The pattern is then transferred to the AlGaAs layer by inductively coupled plasma/reactive ion etching in a Sentech SI500 reactor. Finally, a selective oxidation of the Al-rich layer is performed at 390°C for 40 min, leaving the Al_0.18_Ga_0.82_As metasurface on an AlOx substrate.

### Back focal plane imaging

For our nonlinear experiments we used a Mango optical parametric amplifier from APE pumped with a Satsuma Amplitude laser with 160 fs pulse duration and 500 kHz repetition rate. The wavelength was fixed to 1550 nm, with 21 nm spectral bandwidth, and the polarization was aligned to the [100] crystallographic axis of AlGaAs by a linear polarizer and a half-wave plate. The laser was focused by a 10x microscope objective (NA = 0.3) with optimized transmission at the pump wavelength. A lens with *f* = 200 mm was inserted before the objective to obtain a beam waist radius w_0_ = 50 µm. The reflected pump and SH emitted by the metasurface are collected by the objective before reaching a short-pass dichroic mirror that filters out the pump. A Bertrand lens (*f* = 300 mm) was inserted in a 4 f configuration to perform Fourier space imaging on a CCD camera (Sony, ICX825AL) equipped with a tube lens (*f* = 200 mm).

### Phase characterization

A generalized Mach-Zehnder interferometer, shown in Fig. S6, is used to let the SH generated by the metasurface interfere with a SH reference beam. Half of the pump beam is sent with a beamsplitter on the metasurface to produce a SH vortex. The second half is directed towards a phase-matched BBO to generate a SH reference signal. On this path, a motorized delay line ensures the temporal overlap of the two SH beams that eventually recombine on a second beam splitter. The resulting interference pattern is recorded by the CCD camera. Subsequent Fourier transform can be performed to obtain the phase of the SH signal (see Fig. S7).

### Modal decomposition

The purity of the OAM carrying SH beam is measured by a modal analysis technique described in ref. ^53^ where the SH beam $U(x)$ is decomposed on the Laguerre-Gauss modes $L{G}_{p,n}$ basis of radial order $p=0$, as $U(x,y)={\sum }_{n}{c}_{n}{{\rm{LG}}}_{0,n}(x,y)$. Experimentally, the SH beam emitted by the sample is imaged on a spatial light modulator (200-21, Santec) where each $L{G}_{0,n}$ mode is successively encoded. The modal decomposition coefficients ${\left|{c}_{n}\right|}^{2}$ are determined by measuring the central intensity of the modulated field Fourier transform (Supplementary Section 3.3).

## Supplementary information

Supplemental material
