# Supplementary material for: Unravelling the nonlinear generation of designer vortices with dielectric metasurfaces
Source: Light Sci Appl. 2025 Jan 16;14:51. doi: 10.1038/s41377-025-01741-0 (PMC11739562; doi:10.1038/s41377-025-01741-0)
Supplement: Supplementary file 1 — Supplemental material [file 41377_2025_1741_MOESM1_ESM.docx]

**Supplementary information**

**Unravelling the nonlinear generation of designer vortices with dielectric metasurfaces**

*Laure Coudrat, Guillaume Boulliard, Jean-Michel Gérard, Aristide Lemaître, Aloyse Degiron, Giuseppe Leo**

L. Coudrat, Dr. G. Boulliard, Dr. A Degiron, Prof. G. Leo

Laboratoire Matériaux et Phénomènes Quantiques, Université Paris Cité and CNRS, Paris, 75013, France.

Email: [giuseppe.leo@u-paris.fr](mailto:giuseppe.leo@u-paris.fr)

Dr. Jean-Michel Gérard

Université Grenoble Alpes, CEA, INP, IRIG, PHELIQS, Grenoble, 38000, France.

Dr. A. Lemaître

Centre de Nanosciences et de Nanotechnologies, CNRS - Université Paris-Saclay, Palaiseau, 91120, France.

Prof. G. Leo

Institut universitaire de France (IUF)

Contents

[1 Meta-atom library calculations 3](#_Toc178633667)

[1.1 Influence of the nanoresonator symmetry on the SH far-field angular pattern 3](#_Toc178633668)

[1.2 SH radiation diagram of several resonators used in Fig. 1d. 5](#_Toc178633669)

[1.3 Computed field maps at the FF and SH wavelengths for two orientations of the same half-cylinder 6](#_Toc178633670)

[1.4 Scattering cross-section of an individual half-cylinder resonator suspended in air 7](#_Toc178633671)

[2 Sample fabrication 9](#_Toc178633672)

[3 Experimental characterization 10](#_Toc178633673)

[3.1 Experimental setup 10](#_Toc178633674)

[3.2 SH Phase 11](#_Toc178633675)

[3.3 SH Modal analysis 13](#_Toc178633676)

[3.4 SH generation efficiency 15](#_Toc178633677)

[4 Azimuthal grating and ghost OAM orders 16](#_Toc178633678)

[References 17](#_Toc178633679)

# Meta-atom library calculations

## Influence of the nanoresonator symmetry on the SH far-field angular pattern


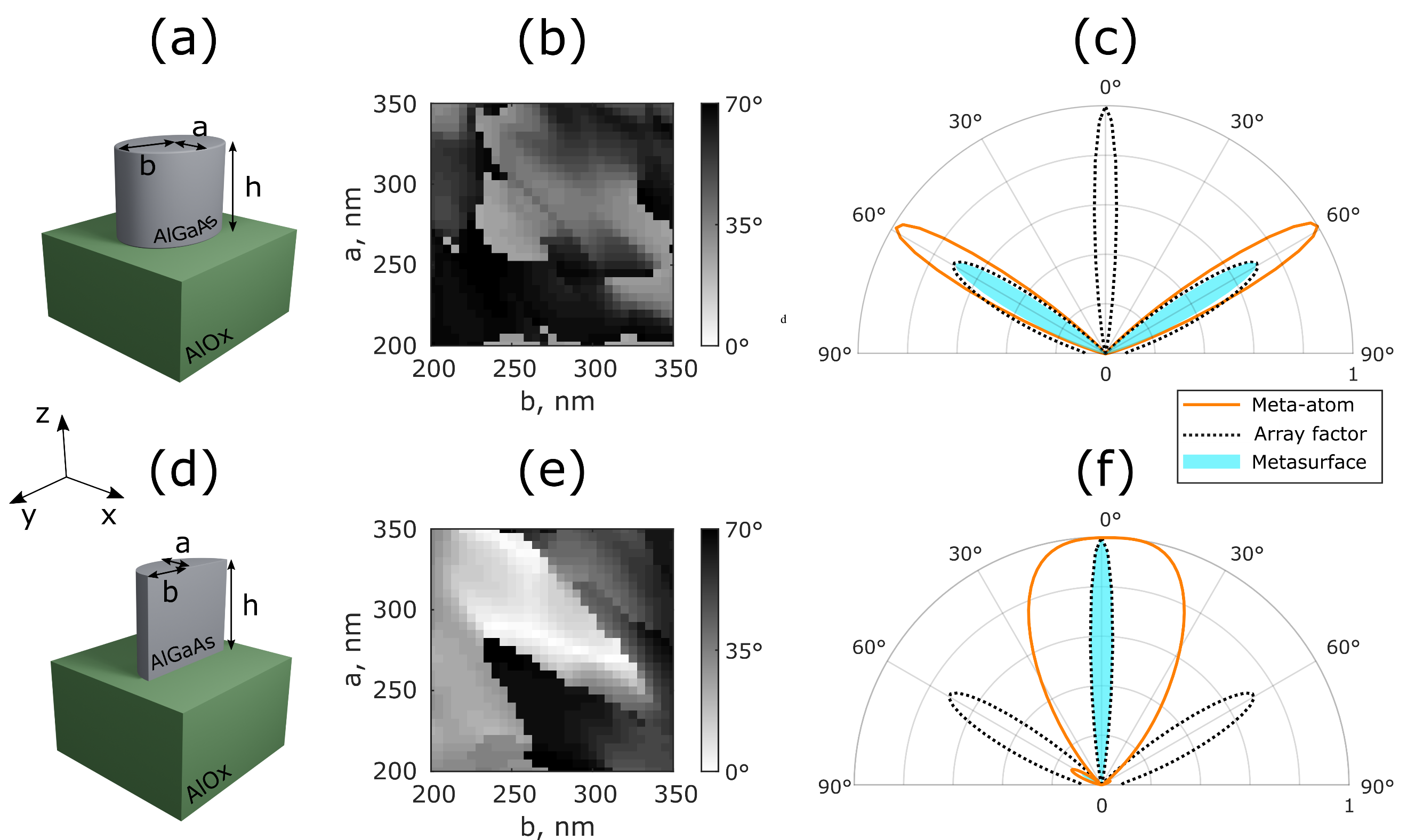


**Fig. S1 Calculated far-field properties of the y component of the SH electric field from [100] AlGaAs nanoresonators.** Simulations are presented for full (a)-(c) and half (d)-(f) cylinders with elliptical base with semi-axes $a$ and $b$ pumped by an x-polarized plane wave at normal incidence. (a), (d) Schematics of the geometry. (b), (e) Angle between the main SHG lobe and the normal to the metasurface. (c), (f) Radiation pattern in the $xz$ plane of the isolated pillar for $a= b=280 nm$ (orange), array factor for a period $\Lambda= 900 nm$ and $N=8$ resonators (dashed black), and product of the two (light blue).

As described in the Methods section of the main text, the SH far-field properties of [100] oriented AlGaAs meta-atoms are calculated as a function of their size parameters with the finite-element method. In Fig. S1, we highlight the SH directionality control enabled by a careful design of the resonators shape. Considering [100] AlGaAs cylinders pumped under normal incidence, the SH emission is oriented at a large angle. The resulting on-axis SH field is null (orange curve in Fig. S1c). However, when the symmetry of the resonator is broken, the direction of the predominant SH emission lobe is along the normal (orange curve in Fig. S1f). This control of the directionality of the SH emission from individual resonators is crucial in metasurface design, since the light generated by each meta-atom is further manipulated through being arrayed. Specifically, the far-field radiation pattern of an array of identical resonators is the product of the resonator radiation diagram and an array factor that reads^1^:

|  | $AF(\hat{r})= \sum_{n=1}^{N} e^{ik\hat{r}.\vec{r}_{n}}$ | (1.1) |
| --- | --- | --- |

where $N$ is the number of resonators, $k$ is the wave number, the unit vector $\hat{r}$ indicates the direction where AF is calculated, and $\vec{r}_{n}$ is the position vector of the $n$th resonator.

For an array period $\Lambda=900$nm and a SH wavelength $\lambda_{SH}=775$ nm, AF exhibits four lobes at oblique incidence and one main lobe along the normal to the surface. The oblique lobes are the (±1,0) and (0,±1) diffraction orders that exist due to the fact that the period is larger than the SH wavelength. With $\Lambda=900$nm and $\lambda_{SH}=775$ nm, these diffraction orders are emitted at 60° from the normal.

For the full cylinders that produce SH emission at large angles, the array factor enhances the emission at oblique incidence, by preferentially funneling the light in the four non-zero diffraction lobes (blue-shaded area of Fig. S1c). In contrast, for half-elliptical cylinders that produce SH emission normal to the surface, the array factor creates an even more directional lobe in this direction (blue-shaded area of Fig. S1f). Note that this conclusion is only true if the metasurface is made of identical resonators. In our vortex-generating metasurfaces, the far-field angular pattern has an annular shape as a consequence of OAM generation.

##
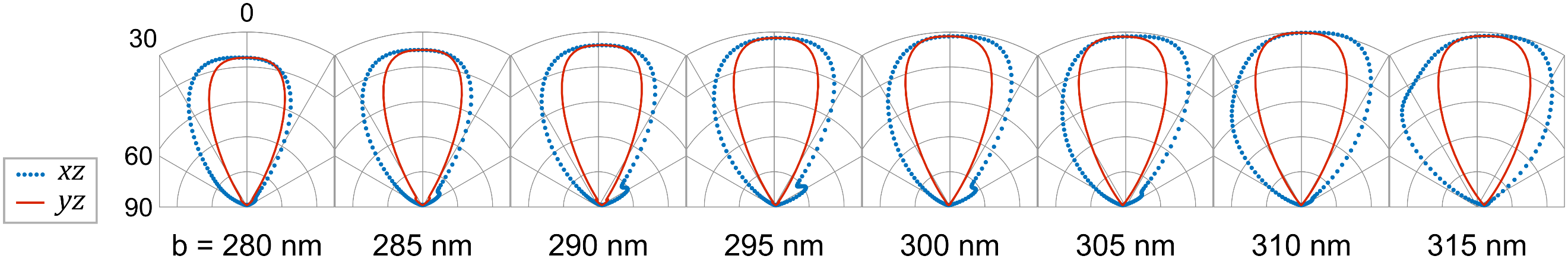
SH radiation diagram of several resonators used in Fig. 1d.

**Fig. S2** SH far-field radiation pattern of individual resonators (i.e. without the AF modulation created by the periodicity) with $a=$ 275 nm and $b$ ranging from 280 to 315 nm.

**Fig. S2** shows the calculated SH far-field radiation pattern of several half-elliptical pillars used in our look-up table.

##
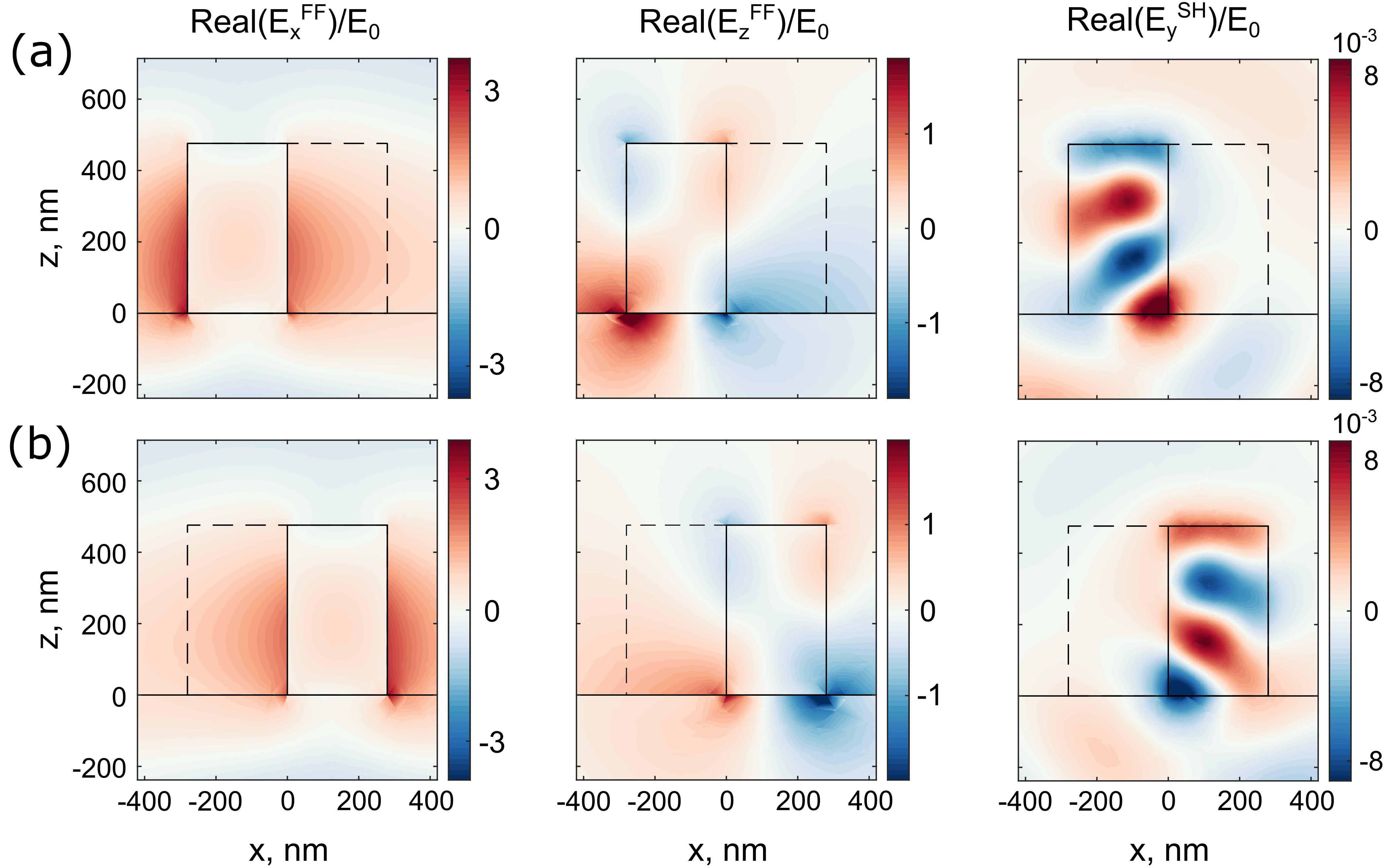
Computed field maps at the FF and SH wavelengths for two orientations of the same half-cylinder

**Fig. S3** Real part of the electric near-field pump x-component (left), z-component (center) and SH y-component (right). Simulations results are shown for the half-elliptical pillar (a) and its π-rotated version about the z-axis (b). Dashed (full) lines indicate the vertical section of a complete (half) elliptic-basis cylindrical resonator on an AlOx substrate. The sizes of the resonator basis are $a = b=$ 280 nm.

The proposed look-up table can be further extended by exploiting the π shift of the z-component of the electric field upon π rotation of the resonator about its vertical axis. Such rotation results in a π shift in the nonlinear polarization $P_{i}^{(2\omega)}=\varepsilon_{0}\sum_{jk} \chi_{ijk}^{\left( 2 \right)}E_{j}^{\omega}E_{k}^{\omega}$ displayed in Fig. S3

##
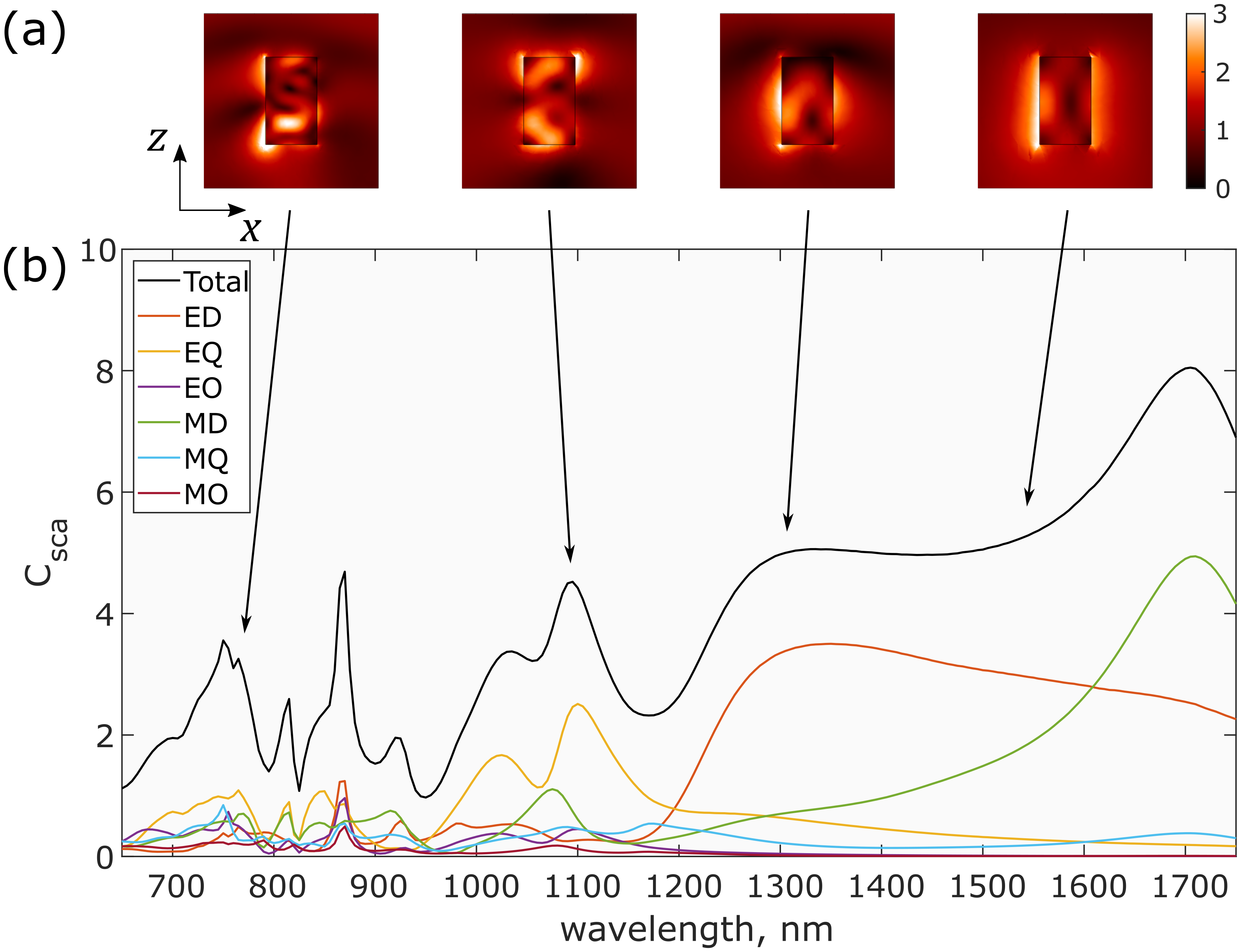
Scattering cross-section of an individual half-cylinder resonator suspended in air

**Fig. S4 Calculated linear response of an isolated half-pillar suspended in air, upon incidence of a plane wave propagating along -z.** (a) Modulus of the electric near-field distributions in the $\boldsymbol{xz}$ plane for several excitation wavelengths, indicated by arrows in b. (b) Linear scattering efficiency and its multipolar decomposition. E: electric, M: magnetic, D: dipole, Q: quadrupole, O: octupole. The dimensions of the pillar are $a=b=280$nm.

Fig. S4 shows the scattering cross-section of an individual half-cylinder resonator, as well as its decomposition in multipoles of increasing order. To perform the multipolar decomposition, we have followed the methodology described in Ref.^2^. Importantly, this methodology can only be applied for resonators surrounded by a uniform medium. This is the reason why we chosen to perform the simulations for a resonator suspended in air. Fig. S4**a** shows the field confinement and the field enhancement inside the resonator volume. The linear scattering efficiency, $C_{sca}$, shown in Fig. S4**b** is the ratio between the scattering cross section of the resonator and its geometric cross section, it shows several resonances including resonances at FF and SH frequencies. The multipolar decomposition of $C_{sca}$, reveals the modes supported by the resonators which are mainly of dipolar nature at the fundamental frequency and quadrupolar at the SH.

# Sample fabrication


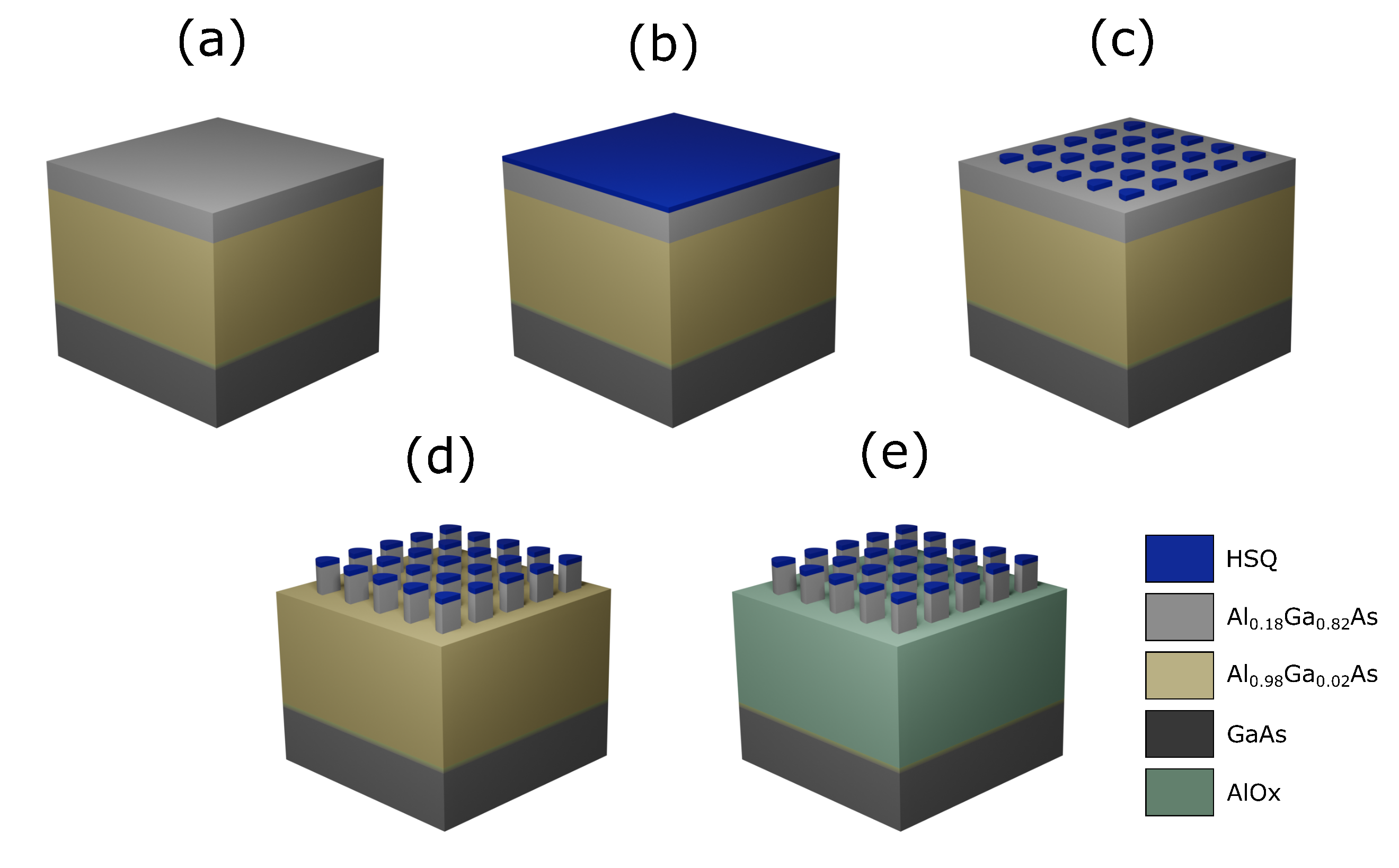


**Fig. S5 Main fabrication steps.** (a) Epitaxial growth. (b) Spin coating of the HSQ resist. (c) Patterning of the metasurface through EBL. (d) Resist pattern transfer through dry etching. (e) Selective oxidation of the aluminum-rich layer.

The fabrication procedure, described in the Materials and Methods section of the main text, is illustrated in Fig. S5.

# Experimental characterization

## Experimental setup


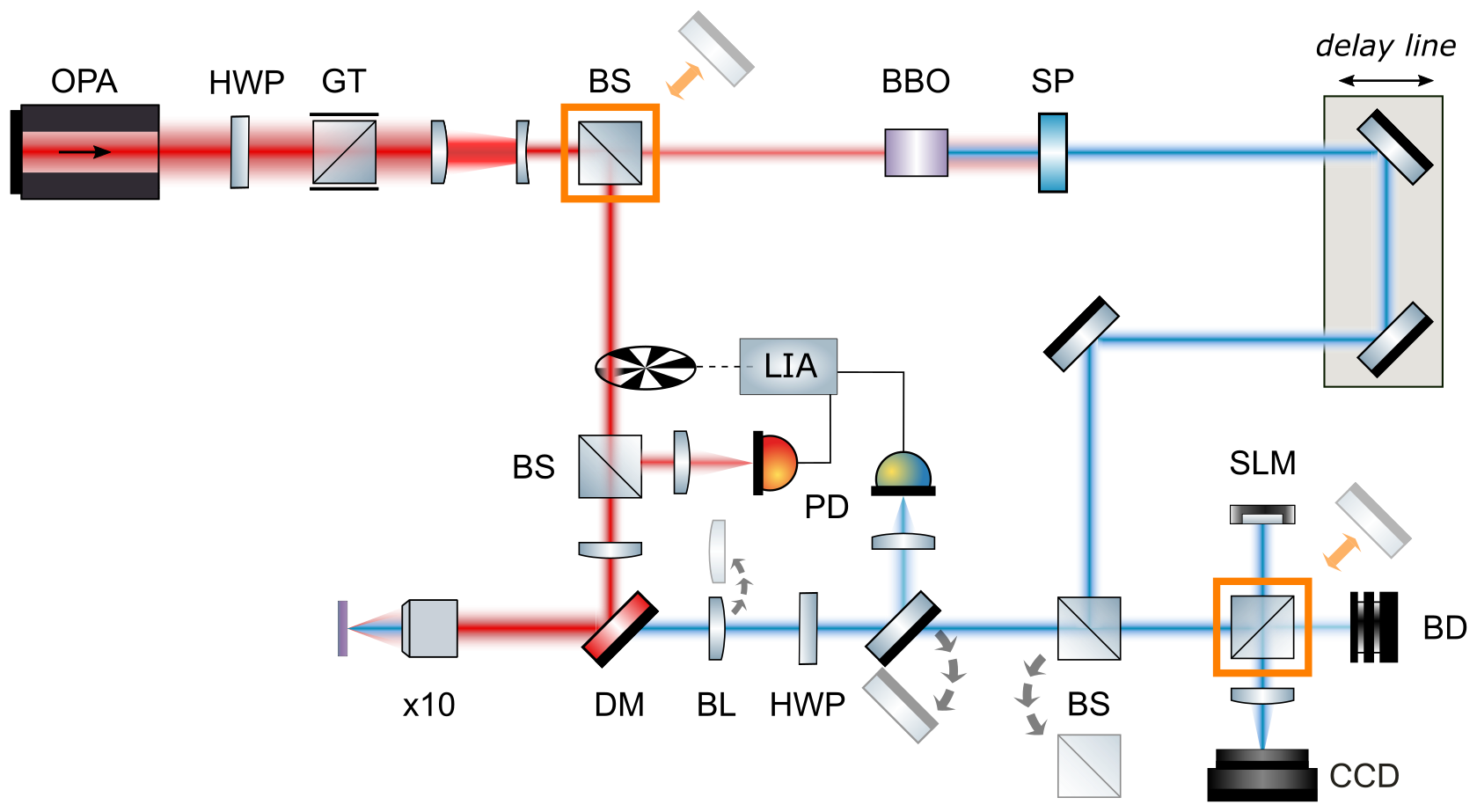


**Fig. S6 Overview of the complete experimental setup for nonlinear metasurfaces characterization.** OPA: Optical Parametric Amplifier, HWP: half-wave plate, GT: Glan Taylor polarizer, LIA: lock-in amplifier, PD: photodiode, DM: dichroic mirror, BL: Bertrand lens, SP: short pass filter, SLM: spatial light modulator, BD: beam dump. The pump and SH beams are represented respectively in red and blue. Orange squares indicate beam splitters (BS) mounted on magnetic bases. They can be replaced for mirrors, making it easy to switch from one configuration to another. The function of each element is explained in the next supplementary figures which focus on sub-parts of this general setup.

The complete SH characterization setup is shown in figure Fig. S6. A mode-locked Yb-doped fiber laser (Satsuma, Amplitude) pumps an optical parametric amplifier (Mango, APE) and provides 160 fs pulses with a repetition rate of 500kHz. Control of the pump power is achieved by a rotating half-wave plate and a Glan-Taylor polarizer. The spot size of the pump beam is reduced in a Galilean telescope. The core of this setup is a home-built microscope. The pump beam is sent by a short-pass dichroic mirror (DMSP950, Thorlabs) and focused by a x10 microscope objective on the surface of the sample. The same objective collects the SH and reflected pump beams before reaching the dichroic mirror that filters out the pump. A Bertrand lens images the back focal plane of the objective, thus allowing to image the Fourier-space SH intensities with a CCD camera (ICX825AL, Sony) equipped with a tube lens. Apart from real and reciprocal space imaging, the set-up implements various functions thanks to an ensemble of kinematic mounts. Beamsplitters can be replaced by mirrors enabling interferometric configuration, modal analysis of the SH beam and efficiency measurements that are described in detail hereafter.

## SH Phase


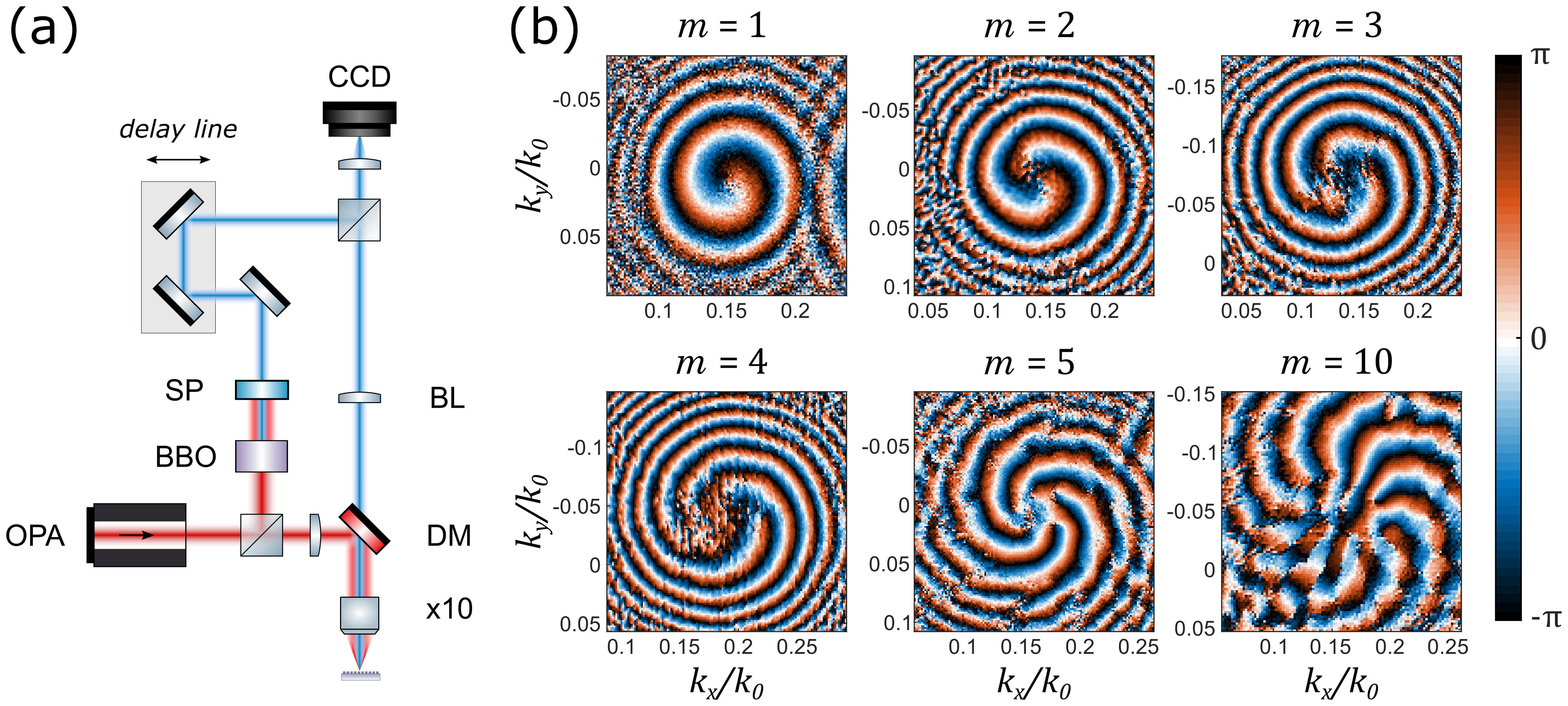


**Fig. S7 SH phase characterization.** (a) Simplified sketch of the setup of Fig. S6, focusing on the Mach- Zehnder interferometry experiment. The first beamsplitter of the interferometer separates the pump at the fundamental wavelength into two beams, while the second beamsplitter recombines the SH beams originating from the metasurface and from a BBO crystal, respectively. OPA: Optical Parametric Amplifier, DM: dichroic mirror, BL: Bertrand lens, SP: short pass filter. The pump and SH beams are represented in red and blue respectively. (b) Phase distributions obtained by numerical Fourier transform of the interference pattern recorded on the CCD.

Optical vortices are associated with their phase texture endowed with a singularity and 0-2π azimuthal variations that must be evidenced experimentally. In this framework, we built a Mach-Zehnder interferometer (Fig. S7**a**) to show the interference pattern between a reference beam and the SH beam emitted by our metasurface, as shown in Fig. 2b and Fig. 3c. This fork-like shape distribution reveals the phase singularity. However, in the case of high topological charges, it may be difficult to resolve the number of fringes spiraling around the fork-dislocation. In this case, the interference pattern can still be used to derive the phase difference between the two beams by implementing a simple post-processing technique. The latter is based on the evaluation of the angle of the digital Fourier transform of the interference pattern centered at the dislocation. This method reveals the phase difference between the two beams as shown in Fig. S7**b**, for the experimental interference pattern of Fig. 2b and Fig. 3c.

## SH Modal analysis

Modal decomposition of the SH vortex beam is performed by measuring the optical overlap of the field emitted by the metasurface, $U(x,y)$, with the modes of the zeroth radial order Laguerre-Gauss basis, $LG_{0,n}$. The experimental implementation, described in ref.^3^, consists in projecting $U(x,y)$ on a SLM where the $LG_{0,n}$ modes are successively encoded. The reflection of $U(x,y)$ on the SLM is Fourier transformed and recorded on a CCD camera, as depicted on the schematic of Fig. S8a. The weights of the different LG modes are directly related to the value of the intensity at the very center of each overlap pattern.


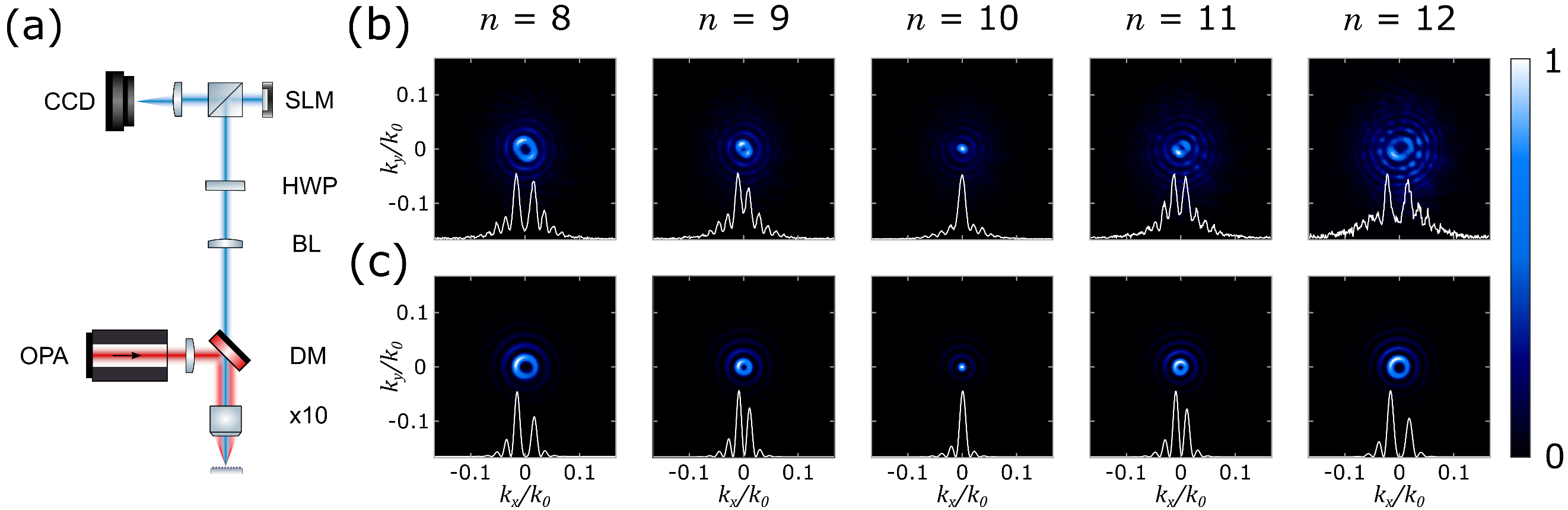


**Fig. S8 Modal analysis.** (a) Simplified sketch of the experimental setup, focusing on the modal decomposition experiment. OPA: Optical Parametric Amplifier, DM: dichroic mirror, BL: Bertrand lens, HWP: half-wave plate, SLM: spatial light modulator. The pump and SH beams are represented in red and blue respectively. (b) Experimental intensity patterns obtained after projecting a SH vortex with $m=10$ (generated by the metasurface of Fig. 2a) on a SLM where modes $LG_{0,n}$ were encoded. (c) Related simulations. The white curves in (b) and (c) are cross-sections of the intensity along the k_x_ axis evaluated at $k_{y}/k_{0}=0$.

Fig. S8b displays the experimental optical overlaps of the SH vortex studied in Fig. 2 ($m=10$) with Laguerre-Gauss modes with azimuthal orders from $n=8$ to $n=12$. Despite an excellent agreement with the simulated patterns, shown in Fig. S8**c**, the intensity in the center of the experimental interference patterns (which is again the region of interest for the modal analysis) does not strictly fall to zero. This difference between the experimental and theoretical patterns can be appreciated more quantitatively by examining the intensity profile along the k_x_ axis (white curves in the same panels). Such small, but non-zero signal in the center of the experimental patterns for $n\neq10$ contributes to increase the weight of the $m\pm1,2$ modes and thus reduces the value of the experimental purity with respect to the calculated one. However, this non-zero signal at the center of the annular overlaps is an experimental artefact that mainly comes from the insufficient spatial resolution of our imaging setup as well as stringent alignment conditions that are extremely difficult to satisfy in practice.


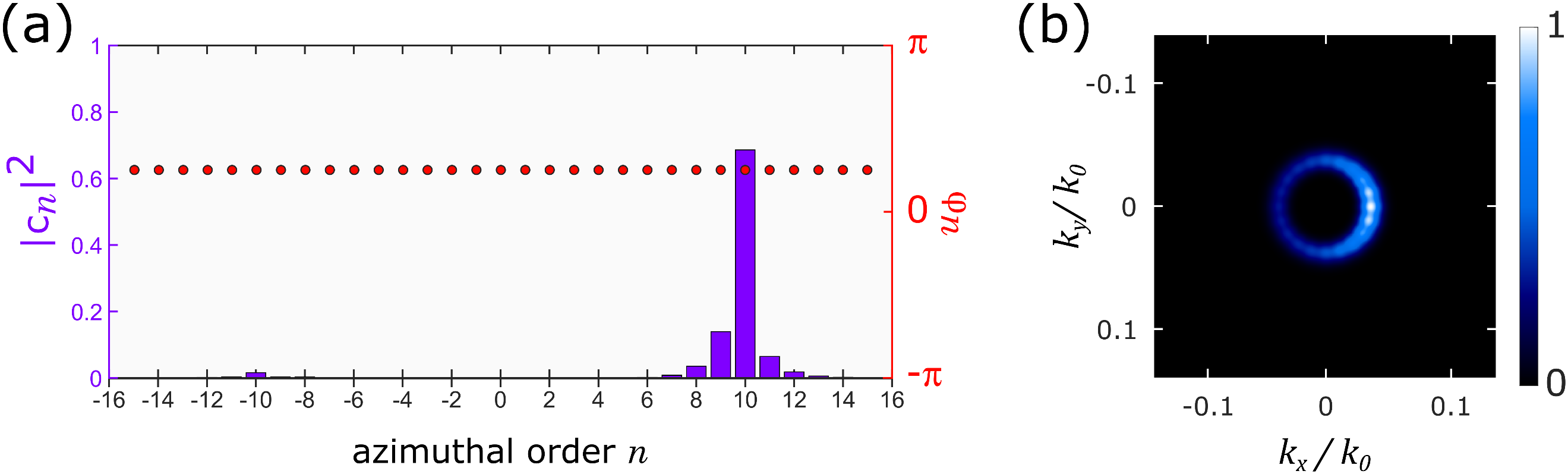
To support this claim, we calculate the reconstructed field intensity $\left| U(x,y) \right|^{2}$ from the experimental modal analysis (Fig. S8b and Fig. S9a) according to $U(x,y)=\sum_{n} c_{n}\mathrm{LG}_{0,n}(x,y)$. The result (Fig. S9b) is an asymmetric intensity profile that does not exhibit the azimuthal undulations predicted by our model and observed experimentally in Fig. 2c. Therefore, we attribute these $m\pm1,2$ contributions to limitations imposed by our instrumentation.

**Fig. S9 SH field reconstruction.** (a) Experimental modal analysis spectrum for the metasurface that generates a SH vortex with $m=10$. These coefficients are derived from the experimental patterns including those displayed in Fig. S8b. The modulus squared $\left| c_{n} \right|^{2}$and the phase $\varphi_{n}$ of the complex weight $c_{n}$ are represented in purple and red, respectively. (b) Intensity of the SH field reconstructed from the modal decomposition of panel a.

##
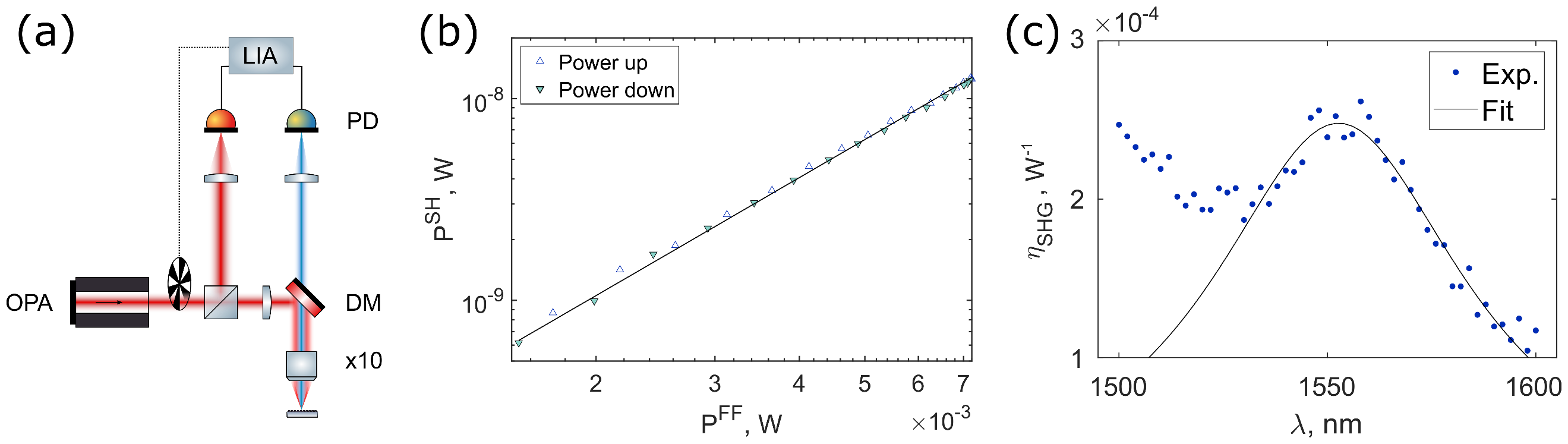
SH generation efficiency

**Fig. S10 Efficiency measurements.** (a) Simplified sketch of the setup of Fig. 3a, focusing on the synchronous detection. OPA: Optical Parametric Amplifier, DM: dichroic mirror, PD: photodiodes, LIA: lock-in amplifier. The pump and SH beams are represented in red and blue respectively. (b) SH time-averaged power versus pump power. Triangles pointing up (down) refer to increasing (decreasing) pump power. (c) Spectral time-averaged normalized efficiency of the metasurface.

Finally, we measured the SH efficiency of the nonlinear metasurfaces. We implemented synchronous detection, depicted on Fig. S10a, for accurate measurement of the SH signal. As would be expected from second order nonlinear processes, the log-log plot of the SH power versus FF power (Fig. S10b) shows a slope close to 2 (1.95). Moreover, we verified the spectral properties of our metasurface by sweeping the pump wavelength between 1.5 and 1.6 µm. The resulting efficiency spectrum (Fig. S10c) shows a resonance peak fitted at 1553 nm, close to the design wavelength at 1550 nm, demonstrating the high-fidelity fabrication process. The FWHM of this resonance is 75 nm (quality factor ~20.5). The peak normalized efficiency defined as $\eta_{SHG}=P_{av}^{SH}/\left( P_{av}^{FF} \right)^{2}\left( RR \tau_{p} \right)$ was estimated to $2.3\times{10}^{-11}W^{-1}$ for a pulsed pump of width 160 fs and 500kHz repetition rate.

#
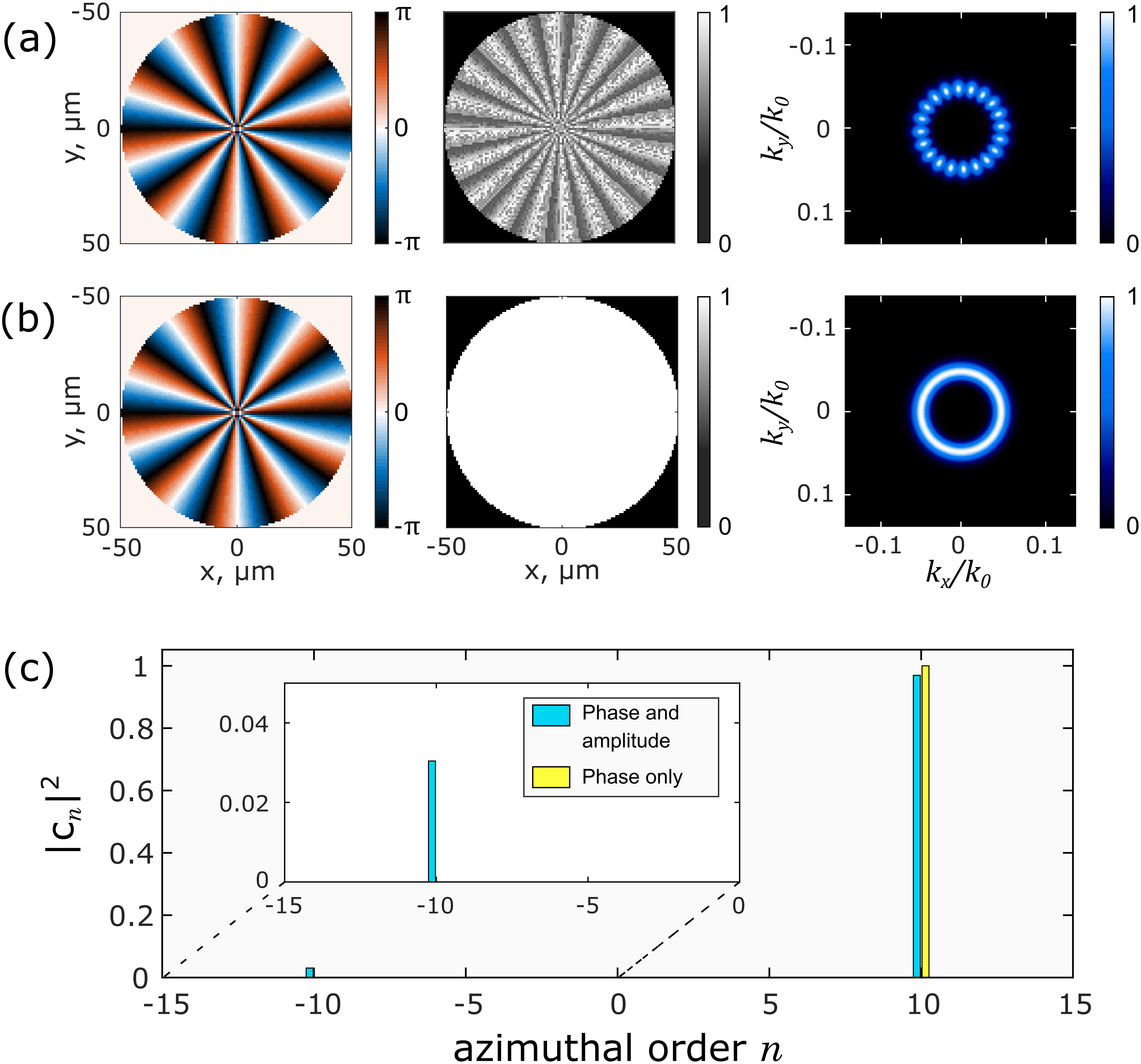
Azimuthal grating and ghost OAM orders

**Fig. S11** **Comparison between the SH vortices generated by our simulated structures, featuring both phase and amplitude variations, and ideal phase-only meta-holograms.** (a) Predictions for our simulated structures. The left and middle panels represent the phase and amplitude variations across the metasurface. These variations reflect the distribution of the resonators that we have picked up in our look-up table to implement the hologram. The predicted Fourier plane intensity, with its typical necklace pattern, is evaluated in the right panel. (b). Same quantities for an ideal phase-only hologram. (c) Calculated modal decomposition for the vortex beams predicted in panel a (blue) and panel b (yellow).

The necklace structure of SH vortices intensity patterns presented in Fig.2c and Fig. 3a, are fully reproduced by our semi-analytical model. The latter predicts the SH field distribution by calculating the far field of the complex hologram $H\left( x,y \right)=A(x,y)\exp(i\varphi(x,y))$, where $A$ and $\varphi$ are respectively the SH amplitude and phase of the resonator at position $(x,y)$ shown in Fig. S11a. A meta-hologram with a strictly constant amplitude results in a perfect annular intensity distribution. Further modal decomposition spectrum in the case of flat amplitude shows the absence of any contributions from the mode with opposite topological charge (Fig. S11b).

# References

1. Balanis, C. A. *Antenna Theory: Analysis and Design*. (Wiley-Interscience, Hoboken, N.J, 2005).

2. Grahn, P., Shevchenko, A. & Kaivola, M. Electromagnetic multipole theory for optical nanomaterials. *New J. Phys.* **14**, 093033 (2012).

3. Pinnell, J. *et al.* Modal analysis of structured light with spatial light modulators: a practical tutorial. *J. Opt. Soc. Am. A* **37**, C146 (2020).
